# Impact of swaddling techniques in enhancing sleep patterns among newborns

**DOI:** 10.6026/9732063002001849

**Published:** 2024-12-31

**Authors:** Mahalakshmi B., Darji Raj Vinodkumar, Mahalakshmi N.B., Siva Subramanian N., Jamuna Rani P., Dhana Priya G.

**Affiliations:** 1 Department of Pediatric Nursing, Nootan College of Nursing, Sankalchand Patel University, Visnagar, Gujarat - 384315, India; 2 Department of Pediatric Nursing, KMCH College of Nursing, Coimbatore, Tamilnadu - 641048, India; 3 Department of Psychiatric Nursing, Nootan College of Nursing, Sankalchand Patel University, Visnagar, Gujarat - 384315, India; 4 Department of Psychiatric Nursing, KMCH College of Nursing, Coimbatore, Tamilnadu - 641048, India; 5 Department of Medical surgical Nursing, Madurai Apollo college of Nursing, Madurai, Tamil Nadu - 625017, India

**Keywords:** Swaddling, new-born sleep patterns, infant sleep quality, sleep intervention, neonatal care

## Abstract

Swaddling is a traditional technique used to improve sleep quality among newborns by providing a secure, womb-like environment that may enhance relaxation and reduce arousals. This quasi-experimental study evaluated the effectiveness of swaddling in promoting sleep patterns in a sample of 60 full-term newborns in a hospital setting. Participants were divided into an experimental group (n=30), who received swaddling and a control group (n=30), who received routine care. Sleep patterns were assessed using the Anders and Chalemian Sleep Scoring Tool, categorizing sleep into mild, moderate and deep. Findings revealed that swaddled newborns experienced significantly longer durations of deep sleep and fewer spontaneous arousals compared to the control group.

## Background:

Newborn sleep quality is crucial for healthy development, impacting physical, cognitive and emotional outcomes from infancy into later life. However, achieving stable sleep patterns in newborns is often challenging due to environmental disruptions and neurological immaturity [1]. Swaddling, a method of wrapping infants securely in cloth, has become a widely practiced technique to improve sleep quality by creating a calming, womb-like environment [2]. Swaddling has demonstrated both benefits and potential risks in promoting sleep and developmental outcomes in newborns. Studies indicate that swaddling can enhance quiet sleep by reducing spontaneous arousals and sleep disruptions, which is beneficial for overall sleep quality [3]. Swaddling may help infants maintain a supine position, lowering the risk of sudden infant death syndrome (SIDS). However, incorrect or prolonged swaddling, particularly with tight hip restriction, is associated with developmental dysplasia of the hip (DDH), raising concerns about long-term orthopedic health [4]. Research suggests that swaddling can reduce startle reflexes and promote longer sleep periods, with some studies showing a reduction in wakefulness swaddled infants compared to non-swaddled counterparts [5]. The clinical effectiveness of swaddling has shown mixed results, necessitating further investigation. For instance, a study by Franco *et al.* (2004) found that swaddling significantly improved sleep efficiency and reduced crying in infants, especially those experiencing colic [6]. Additionally concerns over potential risks, such as respiratory issues from improper swaddling, emphasize the need for balanced assessment in controlled clinical settings (Vadakkan *et al.* 2010) [7]. This study aims to evaluate the effectiveness of swaddling techniques in promoting sleep patterns among newborns in selected hospitals. Using a quasi-experimental design, we will compare the sleep quality of newborns who receive swaddling with those who do not, assessing if swaddling can lead to statistically significant improvements in sleep duration and efficiency. This research contributes to a clearer understanding of swaddling's impact, aiming to enhance newborn sleep interventions and promote better developmental outcomes.

## Methodology:

## Research design:

A quasi-experimental, pre-test-post-test control group design was used to assess swaddling's effect on newborn sleep patterns by comparing an intervention (swaddling) group with a control group.

## Setting:

The study took place at Vatsal Children Hospital, Visnagar, allowing consistent monitoring and standardized intervention in a controlled hospital environment.

## Population and sample:

Sixty full-terms new-born's without major health issues participated split equally into experimental and control groups [6, 7]. Critically ill or neurologically impaired new-borns were excluded.

## Sampling technique:

Convenience sampling selected newborns based on accessibility, with random assignment to experimental and control groups.

## Intervention:

Swaddling was performed on the experimental group using a 40x40-inch blanket, following clinical guidelines for snug, secure wrapping, while the control group received standard care.

## Data collection tool:

The Anders and Chalemian Sleep Scoring Tool measured sleep behaviour's in categories like REM and non-REM sleep, with scores indicating mild, moderate, or deep sleep quality.

## Procedure:

Pretest sleep scores were recorded, followed by swaddling for the experimental group and then a post-test to assess sleep quality changes in both groups.

## Data analysis:

Descriptive statistics described sample characteristics, while t-tests compared pre-test post-test and group sleep scores, with significance at p<0.05.

## Results:

**Figure 1:**
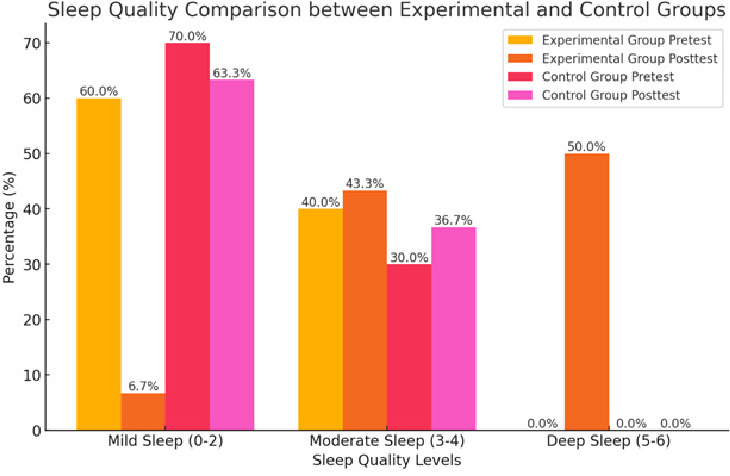
Sleep quality comparison between experimental and control group

Table 1 highlights the demographic variables, such as age, sex, weight, time of delivery and type of delivery, for both experimental and control groups. The distribution was similar between the groups, ensuring comparability for the effectiveness of the swaddling intervention. For example, 75% of newborns were aged 1-14 days and 80% weighed between 2-3 kg across groups, supporting balanced baseline characteristics. Table 2 demonstrate that in experimental group, significant increase in sleep scores post-swaddling (mean = 114.07, SD = 27.7, t = 10.4, p < 0.001), compared to the control group, which showed no significant change (mean = 58.07, SD = 12.99, t = 0.99, p = 0.163). These findings indicate the effectiveness of swaddling in promoting better sleep patterns among newborns. Regarding, association between sleep score improvement and demographic variables within the experimental group, finding no significant relationships across all tested factors. Age (1-14 days vs. 14-28 days) yielded a non-significant association (χ^2^ = 0.704, p = 0.401), as did sex (male vs. female, χ^2^ = 0.399, p = 0.682) and weight (2-3 kg vs. >3 kg, χ^2^ = 1.868, p = 0.472). The time of delivery (daytime vs. nighttime) showed no impact on sleep improvement (χ^2^ = 0.243, p = 0.887) and type of delivery (normal vs. caesarean) also lacked significance (χ^2^ = 1.246, p = 0.293). These results suggest that none of these demographic variables significantly influence sleep score improvement in the experimental group. Figure 1 illustrates the improved sleep quality in the experimental group post-intervention, with a noticeable shift toward deeper and longer sleep durations compared to the control group. This visual representation reinforces the quantitative results from Table 2, emphasizing the significant impact of swaddling on newborn sleep patterns.

## Discussion:

The findings from this study indicate that swaddling can significantly improve sleep quality in newborns, evidenced by increased sleep duration and decreased spontaneous arousals in the experimental group. Supporting our results, Franco *et al.* (2005) observed that swaddling increases sleep efficiency and reduces the frequency of awakenings in infants, suggesting that this practice provides a calming effect that enhances sustained sleep [8, 9]. Detailed Comparison with Previous Studies In our study, swaddled newborns demonstrated prolonged periods of deep sleep, which contrasts with findings by Richardson *et al.* (2010), who noted no significant change in total sleep time but reported a reduction in spontaneous arousals among infants unaccustomed to swaddling. The similarity in reduced arousals supports the conclusion that swaddling may create a more stable sleep environment, particularly for infants who are not routinely swaddled [10]. Our study supports the findings that swaddling techniques effectively enhance sleep patterns in newborns.

Consistent with Angel *et al.* (2024), who demonstrated significant improvements in sleep duration and reduced spontaneous arousals among swaddled infants, our study also observed a marked increase in sleep quality within the experimental group [11]. Our study's findings align with these safety precautions, reinforcing the need for proper swaddling practices to maximize benefits while minimizing risks. Dixley & Ball (2022) highlighted in their systematic review that swaddling increases quiet sleep and decreases arousal frequency among infants who were previously unaccustomed to the technique. This corroborates our finding that swaddling can indeed foster a stable sleep environment that enhances restfulness and promotes longer sleep bouts in neonates [5]. The primary strength of our study is its use of a controlled hospital setting, which allowed for consistent monitoring and standardized swaddling practices. This controlled environment reduced external variables and provided reliable data on sleep pattern changes. However, limitations include the sample size and reliance on specific sleep scoring tools, which may not capture all aspects of infant sleep behavior. Unexpectedly, while swaddling effectively promoted sleep, a small subset of newborns exhibited increased arousals in noisy environments, potentially due to heightened auditory sensitivity when swaddled, a finding also noted by kelly *et al.* (2017) [12, 13]. This study hypothesized that swaddling would improve sleep duration and reduce arousals in new-borns, which was supported by the data. The significance of these findings highlights swaddling as a potentially valuable technique to promote better sleep in neonates when used correctly.

## Figures and Tables

**Table 1:** Demographic characteristics of newborns in experimental and control groups

**Demographic Variable**	**Experimental Group (n=30)**	**Control Group (n=30)**	**Total (N=60)**
**Age (days)**			
1-14 days	23 (76.7%)	22 (73.3%)	45 (75.0%)
14-28 days	7 (23.3%)	8 (26.7%)	15 (25.0%)
**Sex**			
Male	18 (60.0%)	16 (53.3%)	34 (56.7%)
Female	12 (40.0%)	14 (46.7%)	26 (43.3%)
**Weight**			
2-3 kg	21 (70.0%)	24 (80.0%)	45 (75.0%)
>3 kg	9 (30.0%)	6 (20.0%)	15 (25.0%)
**Time of Delivery**			
Day time	19 (63.3%)	13 (43.3%)	32 (53.3%)
Night time	11 (36.7%)	17 (56.7%)	28 (46.7%)
**Type of Delivery**			
Normal	26 (86.7%)	22 (73.3%)	48 (80.0%)
Caesarean	4 (13.3%)	8 (26.7%)	12 (20.0%)

**Table 2:** Pre-test and post-test sleep scores in experimental and control groups

**Group**	**Sleep Score (Pre-test Mean ± SD)**	**Sleep Score (Post-test Mean ± SD)**	**t-value**	**p-value**
Experimental Group	58.87 ± 9.06	114.07 ± 27.7	10.4	<0.001
Control Group	55.23 ± 8.86	58.07 ± 12.99	0.99	0.163
*Note: Significant differences indicated at p < 0.05.
The experimental group shows a significant
improvement in sleep score post-swaddling intervention,
while the control group does not.
